# Time trends in contraceptive prescribing in UK primary care 2000–2018: a repeated cross-sectional study

**DOI:** 10.1136/bmjsrh-2021-201260

**Published:** 2021-11-15

**Authors:** Thomas Joshua Pasvol, E Anne Macgregor, Greta Rait, Laura Horsfall

**Affiliations:** 1 The Research Department of Primary Care and Population Health, University College London, London, UK; 2 Centre for Reproductive Medicine, Barts and the London School of Medicine and Dentistry Centre for Neuroscience and Trauma, London, UK

## Abstract

**Background:**

Over the last 20 years, new contraceptive methods became available and incentives to increase contraceptive uptake were introduced. We aimed to describe temporal trends in non-barrier contraceptive prescribing in UK primary care for the period 2000–2018.

**Methods:**

A repeated cross-sectional study using patient data from the IQVIA Medical Research Data (IMRD) database. The proportion (95% CI) of women prescribed non-barrier contraception per year was captured.

**Results:**

A total of 2 705 638 women aged 15–49 years were included. Between 2000 and 2018, the proportion of women prescribed combined hormonal contraception (CHC) fell from 26.2% (26.0%–26.3%) to 14.3% (14.2%–14.3%). Prescriptions for progestogen-only pills (POPs) and long-acting reversible contraception (LARC) rose from 4.3% (4.3%–4.4%) to 10.8% (10.7%–10.9%) and 4.2% (4.1 %–4.2%) to 6.5% (6.5%–6.6%), respectively. Comparing 2018 data for most deprived versus least deprived areas, women from the most deprived areas were more likely to be prescribed LARC (7.7% (7.5%–7.9%) vs 5.6% (5.4%–5.8%)) while women from the least deprived areas were more likely to be prescribed contraceptive pills (20.8% (21.1%–21.5%) vs 26.2% (26.5%–26.9%)). In 2009, LARC prescriptions increased irrespective of age and social deprivation in line with a pay-for-performance incentive. However, following the incentive’s withdrawal in 2014, LARC prescriptions for adolescents aged 15–19 years fell from 6.8% (6.6%–7.0%) in 2013 to 5.6% (5.4%–5.8%) in 2018.

**Conclusions:**

CHC prescribing fell by 46% while POP prescribing more than doubled. The type of contraception prescribed was influenced by social deprivation. Withdrawal of a pay-for-performance incentive may have adversely affected adolescent LARC uptake, highlighting the need for further intervention to target this at-risk group.

## Introduction

In the UK, approximately 26% of women aged 16–49 years use hormonal contraception.^1^ Several new hormonal methods have become available during the last 20 years, including the desogestrel progestogen-only pill (POP), combined oral contraceptive pills (COCPs) containing drospirenone, combined hormonal patches and vaginal rings. Additionally, the UK has seen a number of policy-related initiatives aimed at reducing unwanted pregnancy.^2 3^

In 2005, the National Institute for Health and Care Excellence (NICE) published its first long-acting reversible contraception (LARC) guideline advising that all women requiring contraception should be given information about LARC.^4^ In 2009, a pay-for-performance Quality and Outcomes (QOF) incentive for LARC counselling was introduced. This incentive aimed to increase LARC uptake by paying general practitioners (GPs) a premium for providing information relating to LARC to women attending for contraception.^3^ Despite its success, the incentive was retired in 2014, and at the same time funding to sexual and reproductive health (SRH) services was reduced.^5^

Detailed data on trends in contraceptive provision from SRH services is published annually by NHS Digital.^6^ However, the majority of women seek contraception from primary care,^7^ with only 5% of females aged 13 to 54 years using SRH service for contraception between 1 April 2019 and 31 March 2020.^6^ Data on contraceptive prescriptions issued in primary care in England are reported in absolute numbers but they are not linked to individual patients nor is data available for the devolved nations of the UK.^6^

Describing trends in contraceptive prescribing and how they relate to demographic factors such as age and deprivation is an essential step in planning future service delivery as the model of contraceptive care undergoes change. We aimed to investigate sociodemographic and temporal trends in the prescribing of non-barrier contraception in primary care from 2000 to 2018.

## Methods

### Study design

A repeated cross-sectional study using electronic UK general practice (GP) records from the IQVIA Medical Research Data (IMRD) database

### Data source

In the UK National Health Service (NHS), GPs look after patients in the community and are often the first point of contact for anyone with a health problem. IMRD is a longitudinal database containing the anonymised medical records of 18.3 million patients across 797 UK GP practices. IMRD represents approximately 6% of the UK population and goes back to 1994. Data are recorded using the Read code hierarchical coding system.^8^ The GP practices included in IMRD are broadly representative of the UK in terms of practice size, age, gender, mortality and the prevalence of a number of chronic conditions such as diabetes, epilepsy and asthma.^9^ IMRD incorporates data from THIN, a Cegadim Database. Reference made to THIN is intended to be descriptive of the data asset licensed by IQVIA.

### Study population

#### Source cohort

First, a source cohort of women was extracted from IMRD. All women aged 15–49 years who contributed data to IMRD for the period 1 January 2000 to 31 December 2018 were eligible for inclusion. This was a dynamic cohort, with women entering and exiting throughout the study period. The age range 15–49 years was selected as this is the World Health Organization (WHO) definition of ‘women of reproductive age’.^10^ All data included were from time periods after the GP practices had met electronic data quality standards.^11 12^

Women were censored from the cohort at the first recording of any medical event which would usually preclude future use of contraception (hysterectomy, bilateral salpingo-oophorectomy or sterilisation), the first recording of a prescription for hormone replacement therapy (online supplemental code lists), the date they de-registered from the practice or the date of death.

#### Repeated-cross sectional data

Separate cross-sections were then identified for each calendar year (2000–2018). To be included, each woman was required to contribute data to the source cohort for the entire year from 1 January to 31 December. A woman could contribute data to multiple cross-sections. In each cross-section, a woman’s age was defined as the age she would be on 1 July of that year (ie, the midpoint of the year).

### Outcomes

The main outcome of interest was the prescription of non-barrier contraceptives. Prescription code lists for the following contraceptives were developed and reviewed by a GP: combined hormonal contraception (CHC) (COCPs, ethinylestradiol and cyproterone acetate (co-cyprindiol), transdermal patches and intravaginal rings), POPs and LARC (intramuscular injections, subdermal implants, intrauterine systems (IUSs) and intrauterine devices (IUDs)). For LARC, Read codes were also used to search the medical records for documented evidence of administration/insertion (online supplemental code lists). Due to a number of non-specific Read codes for IUD/IUS such as ‘reinsertion of coil’, these two contraceptives were grouped together.

COCPs were stratified by pill generation. Pill generation is the four-level UK classification system used for COCPs as they were rolled out chronologically. The majority of pills contain ethinylestradiol and the difference between the generations is the formulation of the progestogen. First-generation pills were not included in the study as they had all been discontinued in the UK by the early 1990s. Co-cyprindiol, a treatment for acne and also a contraceptive, was included separately. Desogestrel 75 *μ*g was separated from other POPs as it works in a similar way to COCPs by inhibiting ovulation.

### Independent variables

The following data were captured for each patient: age (in 5-year bands), country of GP practice, Townsend score (a postcode-linked quintile measurement of deprivation which was taken from the patient’s home address at GP registration. ‘Townsend 1’ is the least deprived and ‘Townsend 5’ is the most deprived).^13^

### Analysis

Stata Statistical Software: Release 15 (2017; StataCorp LLC, College Station, TX, USA) was used for all analyses.

Descriptive characteristics were summarised using numbers and percentages for categorical variables and medians and interquartile ranges (IQRs) for non-normally distributed continuous variables.

The number of women who received each type of contraceptive was reported as a proportion (95% confidence interval (95% CI)) of the total number of women in the cross-section for each year. Multiple prescriptions of the same method within a year were treated the same as a single prescription. Women could be prescribed multiple different types of contraception within 1 year. Proportions were stratified by age group, country and deprivation.

### Patient and public involvement

Patients and the public were not involved in this study.

### Ethics

IMRD data collection was approved by the NHS South-East Multicentre Research Ethics Committee in 2003. This study was approved by the Scientific Research Committee (SRC) on 11 May 2021 (SRC reference 18THIN082-A1).

## Results

### Demographics

A total of 3 577 421 women were included in the source cohort. Nineteen cross-sections were identified, one for each calendar year. 2 705 638 women (15 251 805 person-years) contributed cross-sectional data (table 1). There was minimal difference in median age between cross-sections (range 32.5–33.5 years). Median size of each yearly cross-section was 869 844 (range 4 58 446–9 95 579) patients. Townsend data were missing in 561 233 (20.7%) patients. There was minimal difference in demographics after exclusion of those with missing data (table 1).

### Overall trends

**Figure 1:**
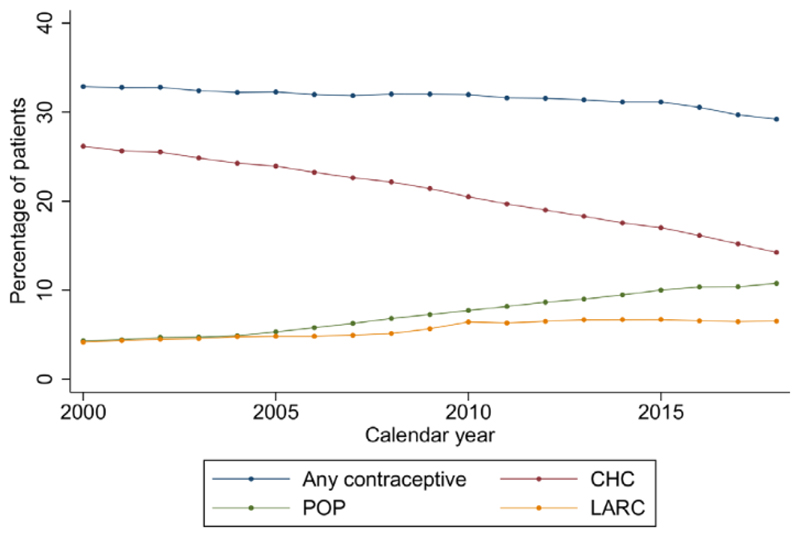
Temporal trends in all non-barrier contraceptive prescribing over the period 2000–2018. CHC, combined hormonal contraception; LARC, long-acting reversiblel contraception; POP, progestogen-only pill.

Between 2000 and 2018, the proportion of women receiving a prescription for any contraceptive fell from 32.9% (32.7%–33.0%) to 29.2% (29.1%–29.3%). However, this was in the context of a rise in prescription of LARC from 4.2% (4.1%–4.2%) to 6.5% (6.5%–6.6%) and POPs from 4.3% (4.3%–4.4%) to 10.8 (10.7%–10.9%) and a fall in prescription of CHCs from 26.2% (26.0%–26.3%) to 14.3% (14.2%–14.3%) (figure 1).

### Combined hormonal contraception

**Figure 2:**
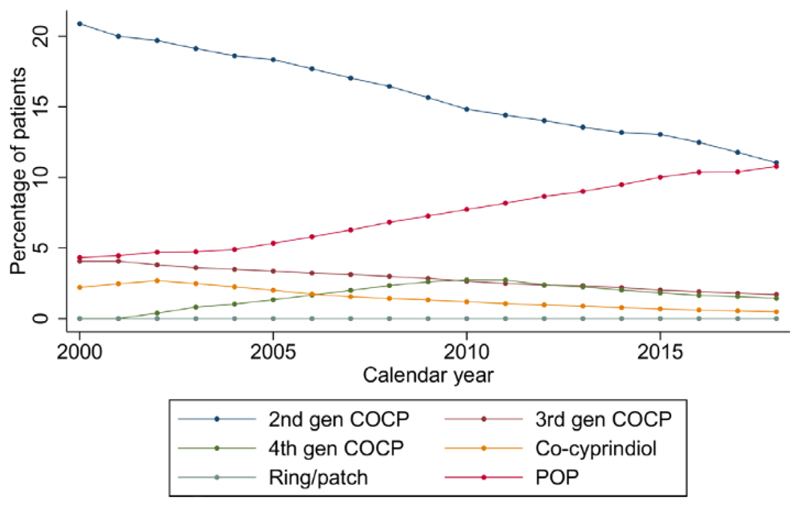
Temporal trends in combined hormonal contraception and progestogen-only pill prescribing over the period 2000–2018. COCP, combined oral contraceptive pill; POP, progestogen-only pill.

Second-generation COCP, third-generation COCP and co-cyprindiol prescriptions fell from 20.9% (20.8%–21.0%) to 11.0% (11.0%–11.1%), 4.1% (4.0%–4.l%) to 1.7% (1.7%–1.7%) and 2.2% (2.2%–2.3%) to 0.5% (0.5%–0.5%), respectively. Fourth-generation COCP prescriptions increased from 0.0% to 2.7% (2.7%–2.8%) in 2010 and then declined to 1.4% (1.4%–1.5%) in 2018. Less than 0.1% were prescribed intravaginal rings and contraceptive patches throughout (figure 2).

Prescribing of CHCs fell in all countries. The largest fall was seen in England, falling from 26.5% (26.3%–26.6%) to 13.8% (13.6%–13.9%) and the smallest fall in Northern Ireland; 24.8% (24.1%–25.6%) to 16.8% (16.4%–17.1%) of women (online supplemental figure 1). CHCs were more commonly prescribed in less deprived areas; 26.5% (26.2%–26.9%) and 21.2% (20.9%–21.5%) in least deprived versus most deprived in 2018, respectively (online supplemental figure 2). Women in their twenties saw the most dramatic reduction in CHC prescribing over the study period, falling from 47.0% (46.6%–47.5%) to 25.2% (24.9%–25.5%) in those aged 20–24 years and 42.8% (42.4%–43.2%) to 20.0% (19.7%–20.3%) in those aged 25–29 years (online supplemental figure 3).

### Progestogen-only pills

Desogestrel prescriptions increased from 0.0% in 2000 to 10.0% (95% CI 9.9 to 10.1) in 2018 (desogestrel 75 *μ*g was introduced in 2002), whereas prescription of other POPs fell from 4.3% (4.3%–4.4%) to 1.0% (0.9%–1.0%). Prescribing of POPs rose in all countries, the most dramatically in Northern Ireland from 3.2% (2.9%–3.5%) to 11.6% (11.3%–11.9%) (online supplemental figure 4). A similar increase in POP prescribing was observed across all age and socioeconomic groups (online supplemental figure 5 and 6).

### Long-acting reversible contraception

**Figure 3:**
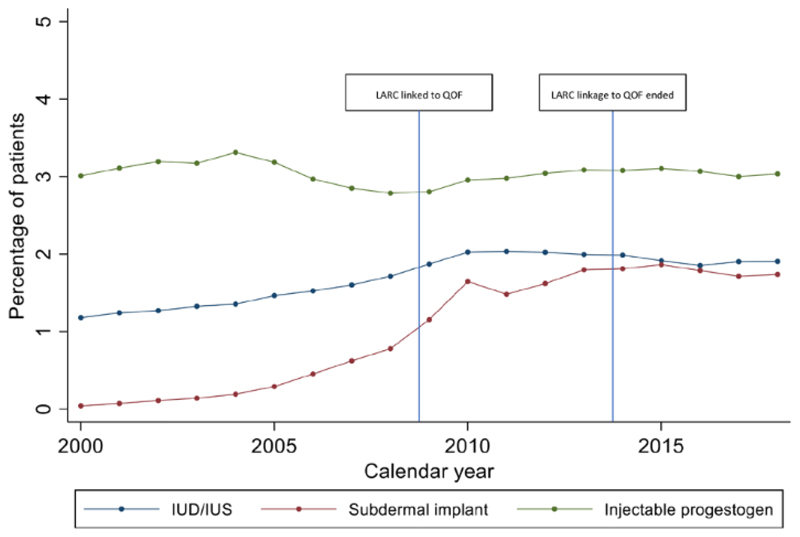
Temporal trends in long-acting reversible contraceptive prescribing over the period 2000–2018. IUD, intrauterine device; IUS, intrauterine system.

IUD/IUS prescribing increased from 1.2% (1.1%–1.2%) in 2000 to 1.9% (1.9%–1.9%) in 2018. Implant prescribing increased from 0.0% to 1.7% (1.7%–1.8%); the older levonorgestrel implants were discontinued and replaced by etonogestrel implants in 1999. IUD/IUS and implant uptake increased more rapidly in line with LARC linkage to QOF in 2009 and plateaued after this date (figure 3). Injectable contraception prescribing was fairly constant throughout the study period fluctuating from 2.8% (2.8%–2.8%) to 3.3% (3.3%–3.4%). However, injectable prescribing fell during the period 2005–2009, then rose marginally when LARC was linked to QOF in 2009 (figure 3). After the pay-for-performance QOF ended in 2014, LARC prescribing fell from 6.7% (6.6%–6.7%) in 2013 to 6.5% (6.5%–6.6%) in 2018 (figure 1).

All countries saw a rise in uptake of LARC over the study period; the largest in Scotland from 4.6% (4.4%–4.8%) to 8.0% (7.9%–8.2%) and the smallest in Northern Ireland from 3.6% (3.3%–3.9%) to 4.8% (4.6%–5.0%) (online supplemental figure 7). LARC was more commonly prescribed in areas of greater deprivation 5.6% (5.4%–5.8%) and 7.7% (7.5%–7.9%) for least deprived versus most deprived, respectively, in 2018 (online supplemental figure 8).

Adolescents aged 15–19 years and women aged 20–24 years saw the biggest increase in LARC prescribing between 2000 and 2013; from 3.9% (3.7%–4.1%) to 6.8% (6.6%–7.0%) and 6.1% (5.9%–6.3%) to 8.9% (8.7%–9.0%), respectively. The only age group to see a reduction in LARC prescription after linkage to QOF ended was adolescents, falling from 6.8% (6.6%–7.0%) in 2013 to 5.6% (5.4%–5.8%) in 2018 (online supplemental figure 9).

## Discussion

Over a 19-year period, prescription of CHCs almost halved, but a 2.5-fold increase in POP prescription was found. LARC prescription increased in line with the introduction of the NICE guidance in 2005, with a further increase following the pay-for-performance QOF indicator in 2009. Adolescents were the only age group to see a fall in LARC uptake after the QOF indicator was withdrawn in 2014.

Strengths of this study include the large sample size and the use of a database generalisable to the UK population. Unlike survey studies which rely on self-reporting of contraceptive use, our data are based on prospectively collected electronic prescribing records, thus avoiding recall bias.

There are a number of limitations to this study. First, contraceptive prescriptions from SRH services were not included. Additionally, some GPs do not offer implant or IUD/IUS insertion and women will have to obtain these elsewhere. Therefore, results are an underestimate of actual contraceptive uptake, but still an accurate representation of prescribing in primary care. Second, it is acknowledged that a proportion of women will have been prescribed methods for non-contraceptive reasons (eg, IUS for menorrhagia). Third, although we captured LARC insertions, some of these devices can remain in situ for up to 10 years. Therefore, we are not able to provide reliable estimates of prevalent use for these methods. Finally, although IMRD has been shown to be generalisable to the UK population, when stratifying by country, contributing practices are not necessarily generalisable to the region.

Similarly to the USA, Ireland, Australia and Canada, oral contraception was the most commonly prescribed method.^14–17^ We observed comparable estimates of prescribing to a population-based Northern Irish study for the period 2010–2016 (20.2% vs 16.6% for COCPs and 9.4% vs 8.0% for POPs).^18^ The small difference could be explained by the fact that these researchers were unable to link 11% of dispensed contraceptives to individuals and these were not included. In comparison to a Clinical Practice Research Datalink study focusing on the impact of QOF on LARC uptake for the period 2004–2014, our estimates of LARC prescribing were higher (5.7% vs 3.0% for 2009 and 6.7% vs 3.9% for 2014).^19^ This could be because this research group classified LARC uptake as ‘a branded or generic prescription for LARC’ whereas we additionally included documentation of insertion in the medical records. We observed similar trends in LARC prescribing before and during the period that LARC was linked to QOF. However, our study provides new evidence that LARC prescribing has fallen in adolescents since the QOF indicator was withdrawn in 2014. An explanation could be the fact that young people are more likely to be new users of contraception; new users may be more likely to take up LARC when offered it than women already established on contraception that works for them. Young women are the most at risk of unplanned pregnancy,^20^ and if LARC uptake in adolescents has fallen then this is a concern. A study assessing rates of unplanned pregnancies in relation to withdrawal of the QOF indicator would a useful piece of work. This could guide decision-making regarding re-implementation of the incentive or the introduction of new interventions.

In keeping with data from Canada and Ireland,^14 16^ we found LARC to be more commonly prescribed in deprived areas and oral contraception to be more commonly prescribed in less deprived areas. These trends are likely to be influenced by social inequalities. Contraceptive failure rates have been shown to be higher across all methods in low-income groups.^21^ Additionally, in England and Wales, the rate of abortion in the most deprived decile is more than double the rate in the least deprived.^22^ These factors could generate prejudice among GPs when selecting appropriate contraception. Practitioners must be trained to provide informed contraceptive choices, including appropriate information and education to avoid prescribing inequalities.

An increase in prescribing of POPs was expected since the introduction of desogestrel 75 *μ*g in 2002.^23^ POP prescribing may have also increased due to a shift towards administration of medications via patient group direction; non-medical prescribers may be more likely to supply medications with fewer risks and contraindications.^24^ This would account for the reduction in COCP prescribing mirroring the increase in POP prescription. Recently, desogestrel 75 *μ*g became available over-the-counter.^25^ While this broadens contraceptive availability, desogestrel 75 *μ*g is not as effective as LARC. We hope that pharmacists will use the opportunity to signpost women, particularly adolescents, to information on LARC and reverse the falling trend in this population.

Our study highlights temporal and sociodemographic trends in contraceptive prescribing across the UK. How contraceptive care is delivered is currently in a period of transition. Prescribers will need to be attuned to changes in demand for contraception, so that any evolving model responds to women’s choices and needs.

## Supplementary Material

Supplementary data

## Figures and Tables

**Table 1:** Descriptive characteristics of women contributing cross-sectional data with and without the inclusion of those with missing Townsend score

Characteristic	Primary analysis	Exclusion of women with missing data
Overall (n)	2 705 638	2 144 405
Country (n (%))		
England	1 885 015 (69.7)	1 555 734 (72.6)
Scotland	405 013 (15.0)	310 706 (14.5)
Wales	314 468 (11.6)	201 680 (9.4)
Northern Ireland	101 142 (3.7)	76 285 (3.6)
Townsend, quintile (n (%))		
Missing	561 233 (20.7)	N/A
1 (least deprived)	482 529 (17.8)	482 529 (22.5)
2	427 003 (15.8)	427 003 (19.9)
3	470 492 (17.4)	470 492 (21.9)
4	446 378 (16.5)	446 378 (20.8)
5 (most deprived)	318 003 (11.8)	318 003 (14.8)
Age at cohort entry (years) (median (IQR))	28.0 (20.5–35.8)	28.0 (20.5–36.1)
Cohort follow-up (years) (median (IQR))	4.9 (2.7–9.1)	5.2 (2.9–9.4)

IQR, interquartile range; N/A, not applicable.

## Data Availability

Data are available upon reasonable request. Data may be obtained from a third party and are not publicly available.

## Key messages

▸ Over a 19-year period, prescription of combined hormonal contraception almost halved while progestogen-only prescriptions more than doubled.
▸ Long-acting reversible contraception (LARC) prescriptions were higher in women from most deprived areas while oral contraception prescriptions were higher in women from least deprived areas.
▸ Pay-for-performance incentives to increase LARC prescription were effective, but their withdrawal may have adversely affected adolescent LARC uptake.
