# Supplementary material for: Time trends in contraceptive prescribing in UK primary care 2000–2018: a repeated cross-sectional study
Source: BMJ Sex Reprod Health. 2021 Nov 15;48(3):193–8. doi: 10.1136/bmjsrh-2021-201260 (PMC9279840; doi:10.1136/bmjsrh-2021-201260)
Supplement: Supplementary file 1 [file bmjsrh-2021-201260supp001.pdf]

Supporting information – Prescription and Read code lists

1. Second generation combined oral contraceptive pills

Drug code	Generic name
38631978	Ethinylestradiol 35microgram / Norgestimate 250microgram tablets
42618978	Ethinylestradiol 35microgram / Norgestimate 250microgram tablets
58067979	Ethinylestradiol 30microgram / Levonorgestrel 150microgram tablets
61424979	Ethinylestradiol 35microgram / Norgestimate 250microgram tablets
72983978	Ethinylestradiol 30microgram / Levonorgestrel 150microgram tablets
72984978	Ethinylestradiol 30microgram / Levonorgestrel 150microgram tablets
81388998	Ethinylestradiol 30microgram / Levonorgestrel 150microgram tablets
81713998	Ethinylestradiol 30microgram / Levonorgestrel 150microgram tablets
82039998	Generic Logynon tablets
82040998	Ethinylestradiol 30microgram / Levonorgestrel 150microgram tablets
82343998	Ethinylestradiol 30microgram / Levonorgestrel 150microgram tablets
83562978	Ethinylestradiol 35microgram / Norgestimate 250microgram tablets
89080998	Generic Microgynon 30 ED tablets
89213998	Ethinylestradiol 30microgram / Levonorgestrel 150microgram tablets
90566998	Ethinylestradiol with norethisterone - biphasic 7 x 35mcg+500mcg; 14 x 35mcg+1mg tablet
90641998	Generic Logynon ED tablets
90644998	Generic Logynon tablets
90647998	Levonorgestrel 250microgram / ethinylestradiol 50microgram tablets
90650998	Levonorgestrel 250microgram / ethinylestradiol 30microgram tablets
90654998	Ethinylestradiol 30microgram / Levonorgestrel 150microgram tablets
90658998	Ethinylestradiol & levonorgestrel 50mcg+250mcg tablets
90703997	Ethinylestradiol with norethisterone - triphasic 7 x 35+500mcg; 7 x 35+750mcg; 7 x 35mcg+1mg tablet
90972998	Ethinylestradiol 35microgram / Norgestimate 250microgram tablets
92682998	Mestranol 50microgram / Norethisterone 1mg tablets
92860979	Ethinylestradiol & levonorgestrel 50mcg+250mcg tablets
92862979	Ethinylestradiol 30microgram / Levonorgestrel 150microgram tablets
94158996	Ethinylestradiol 30microgram / Norethisterone acetate 1.5mg tablets
94158997	Ethinylestradiol 20microgram / Norethisterone acetate 1mg tablets
94997992	Ethinylestradiol 30microgram / Levonorgestrel 150microgram tablets
95885998	Mestranol 50microgram / Norethisterone 1mg tablets
97456998	Ethinylestradiol & levonorgestrel 50mcg+250mcg tablets
97462998	Generic Logynon ED tablets
97464998	Ethinylestradiol 30microgram / Levonorgestrel 150microgram tablets
97466998	Ethinylestradiol & levonorgestrel 30mcg+250mcg tablets
97563998	Generic Synphase tablets
98085997	Ethinylestradiol 35microgram / Norethisterone 1mg tablets
98085998	Ethinylestradiol 35microgram / Norethisterone 500microgram tablets
98181997	Ethinylestradiol with norethisterone - triphasic and placebo 7 x 35+500mcg; 7 x 35+750mcg; 7 x 35mcg+1mg tablet
98181998	Generic trinovum tablets
98183998	Ethinylestradiol 35microgram / Norethisterone 500microgram tablets
98185998	Mestranol & norethisterone 50mcg+1mg tablets

98187998	Ethinylestradiol & norethisterone 35mcg+1mg tablets
98189998	Generic binovum tablets
98191998	Mestranol 50microgram / Norethisterone 1mg tablets
98193998	Ethinylestradiol 35microgram / Norethisterone 500microgram tablets
98195998	Ethinylestradiol 35microgram / Norethisterone 1mg tablets
98197998	Generic Logynon tablets
98199998	Ethinylestradiol 30microgram / Levonorgestrel 150microgram tablets
98201998	Ethinylestradiol & levonorgestrel 30mcg+250mcg tablets
98203998	Ethinylestradiol & levonorgestrel 50mcg+250mcg tablets
98205998	Generic Logynon tablets
98207998	Ethinylestradiol 30microgram / Norethisterone acetate 1.5mg tablets
98209998	Ethinylestradiol 20microgram / Norethisterone acetate 1mg tablets
99036998	Ethinylestradiol 35microgram / Norgestimate 250microgram tablets
99047998	Ethinylestradiol 35microgram / Norgestimate 250microgram tablets
89341998	Ethinylestradiol with levonorgestrel 30micrograms + 50micrograms tablet
90703998	Ethinylestradiol with norethisterone - triphasic 7x35+500mcg; 9x35mcg+1mg; 5x35+500mcg tablet
93781998	Ethinylestradiol with levonorgestrel tablet
94995992	Ethinyloestradiol/norethisterone 35 mcg tab
95002992	Ethinylestradiol & levonorgestrel 50mcg+250mcg tablets

2. Third generation combined oral contraceptive pills

Drug code	Generic name
39702978	Ethinylestradiol 30microgram / Desogestrel 150microgram tablets
47222978	Ethinylestradiol 20microgram / Desogestrel 150microgram tablets
52480979	Ethinylestradiol 30microgram / Desogestrel 150microgram tablets
52481979	Ethinylestradiol 20microgram / Desogestrel 150microgram tablets
53192979	Ethinylestradiol 30microgram / Desogestrel 150microgram tablets
59313978	Ethinylestradiol 30microgram / Desogestrel 150microgram tablets
72985978	Ethinylestradiol 30microgram / Desogestrel 150microgram tablets
72986978	Ethinylestradiol 30microgram / Desogestrel 150microgram tablets
82024998	Ethinylestradiol 20microgram / Gestodene 75microgram tablets
82029998	Ethinylestradiol 30microgram / Gestodene 75microgram tablets
82032998	Ethinylestradiol 20microgram / Desogestrel 150microgram tablets
82041998	Ethinylestradiol 30microgram / Desogestrel 150microgram tablets
84491998	Ethinylestradiol 20microgram / Gestodene 75microgram tablets
84492998	Ethinylestradiol 30microgram / Gestodene 75microgram tablets
90747998	Ethinylestradiol 30microgram / Desogestrel 150microgram tablets
90750998	Ethinylestradiol 20microgram / Desogestrel 150microgram tablets
90760998	Generic Femodene ED tablets
90969997	Ethinylestradiol 20microgram / Gestodene 75microgram tablets
90969998	Ethinylestradiol 30microgram / Gestodene 75microgram tablets
92485998	Ethinylestradiol 20microgram / Gestodene 75microgram tablets
92863979	Ethinylestradiol 30microgram / Desogestrel 150microgram tablets
93263998	Generic Femodene ED tablets
94398997	Ethinylestradiol 20microgram / Gestodene 75microgram tablets

94398998	Ethinylestradiol 30microgram / Gestodene 75microgram tablets
94745998	Ethinylestradiol 20microgram / Desogestrel 150microgram tablets
94773998	Ethinylestradiol 30microgram / Gestodene 75microgram tablets
96439997	Ethinylestradiol 30microgram / Desogestrel 150microgram tablets
96439998	Ethinylestradiol 20microgram / Desogestrel 150microgram tablets
96922998	Ethinylestradiol 30microgram / Gestodene 75microgram tablets
98178998	Ethinylestradiol 30microgram / Desogestrel 150microgram tablets
90757998	Ethinylestradiol with gestodene - triphasic 6 x 30+50mcg; 5 x 40+70mcg; 10 x 30+100mcg tablet
97670998	Generic tri-minulet tablets
97702998	Generic tri-minulet tablets

3. Fourth generation combined oral contraceptive pills

Drug code	Generic name
45866978	Ethinylestradiol 20microgram / Drospirenone 3mg tablets
46090978	Ethinylestradiol 20microgram / Drospirenone 3mg tablets
47150978	Ethinylestradiol 30microgram / Drospirenone 3mg tablets
53008979	Ethinylestradiol 20microgram / Drospirenone 3mg tablets
53009979	Ethinylestradiol 20microgram / Drospirenone 3mg tablets
72966978	Ethinylestradiol 30microgram / Drospirenone 3mg tablets
74455978	Ethinylestradiol 30microgram / Drospirenone 3mg tablets
78546978	Ethinylestradiol 30microgram / Drospirenone 3mg tablets
83634998	Ethinylestradiol 20microgram / Drospirenone 3mg tablets
83740978	Estradiol 1.5mg / Nomegestrol 2.5mg tablets
83741978	Estradiol 1.5mg / Nomegestrol 2.5mg tablets
84583978	Ethinylestradiol 20microgram / Drospirenone 3mg tablets
59254978	Ethinylestradiol 30microgram / Drospirenone 3mg tablets
59255978	Ethinylestradiol 30microgram / Drospirenone 3mg tablets
82867998	Generic Qlaira tablets
82869998	Generic Qlaira tablets
89914979	Ethinylestradiol 30microgram / Drospirenone 3mg tablets
92571998	Ethinylestradiol 30microgram / Drospirenone 3mg tablets
98852998	Ethinylestradiol 30microgram / Drospirenone 3mg tablets

4. Co-cyprindiol

Drug code	Generic name
47175978	Co-cyprindiol 2000microgram/35microgram tablets
85864998	Co-cyprindiol 2000microgram/35microgram tablets
86466998	Co-cyprindiol 2000microgram/35microgram tablets
86925998	Co-cyprindiol 2000microgram/35microgram tablets
87351998	Co-cyprindiol 2000microgram/35microgram tablets
90826979	Co-cyprindiol 2000microgram/35microgram tablets
90828979	Co-cyprindiol 2000microgram/35microgram tablets

90833979	Co-cyprindiol 2000microgram/35microgram tablets
91068998	Co-cyprindiol 2000microgram/35microgram tablets
91069998	Co-cyprindiol 2000microgram/35microgram tablets
94832990	Co-cyprindiol 2000microgram/35microgram tablets
94920998	Co-cyprindiol 2000microgram/35microgram tablets
95220990	Co-cyprindiol 2000microgram/35microgram tablets
95396990	Co-cyprindiol 2000microgram/35microgram tablets
96577998	Co-cyprindiol 2000microgram/35microgram tablets
97520998	Co-cyprindiol 2000microgram/35microgram tablets

5. Vaginal rings

Drug code	Generic name
83186998	Ethinylestradiol 2.7mg / Etonogestrel 11.7mg vaginal delivery system
84617998	Ethinylestradiol 2.7mg / etonogestrel 11.7mg vaginal delivery system

6. Contraceptive patches

Drug code	Generic name
85771979	Ethinylestradiol 33.9micrograms/24hours / Norelgestromin 203micrograms/24hours transdermal patches
85772979	Ethinylestradiol 33.9micrograms/24hours / Norelgestromin 203micrograms/24hours transdermal patches
89295998	Ethinylestradiol 33.9micrograms/24hours / Norelgestromin 203micrograms/24hours transdermal patches
91878998	Ethinylestradiol 33.9micrograms/24hours / Norelgestromin 203micrograms/24hours transdermal patches
94918998	Ethinylestradiol 33.9micrograms/24hours / Norelgestromin 203micrograms/24hours transdermal patches

7. Progesterone-only pills

Drug code	Generic name
72965978	Desogestrel 75microgram tablets
53167979	Desogestrel 75microgram tablets
90581998	Desogestrel 75microgram tablets
98172998	Norethisterone 350microgram tablets
83545978	Desogestrel 75microgram tablets
61400979	Desogestrel 75microgram tablets
98170998	Levonorgestrel 30microgram tablets
90580998	Desogestrel 75microgram tablets
97451998	Levonorgestrel 75mcg tablets
95699998	Norgestrel 75microgram tablets
53171979	Desogestrel 75microgram tablets
85168978	Desogestrel 75microgram tablets
97599998	Etynodiol diacetate 500mcg tablets

93986998	Levonorgestrel 30microgram tablets
53168979	Desogestrel 75microgram tablets
83189978	Desogestrel 75microgram tablets
97452998	Levonorgestrel 30microgram tablets
82528978	Desogestrel 75microgram tablets
96765998	Etynodiol 500microgram tablets
91333998	Levonorgestrel 750microgram tablets
93893998	Norethisterone 350microgram tablets
53169979	Desogestrel 75microgram tablets
98174998	Norethisterone 350microgram tablets
53166979	Desogestrel 75microgram tablets

8. Long-acting reversible contraception prescription codes

Drug code	Generic name
20364978	Levonorgestrel 19.5mg intrauterine device
20365978	Levonorgestrel 19.5mg intrauterine device
50916978	Levonorgestrel 20micrograms/24hours intrauterine device
58042979	Intrauterine contraceptive device
58043979	Intrauterine contraceptive device
58044979	Intrauterine contraceptive device
59356979	Intrauterine contraceptive device
59358979	Intrauterine contraceptive device
59359979	Intrauterine contraceptive device
59360979	Intrauterine contraceptive device
71058994	Intrauterine contraceptive device
75898978	Levonorgestrel 13.5mg intrauterine device
75899978	Levonorgestrel 13.5mg intrauterine device
80741994	Intrauterine contraceptive device
83855994	Intrauterine contraceptive device
83856994	Intrauterine contraceptive device
83858994	Intrauterine contraceptive device
83859994	Intrauterine contraceptive device
84171994	Intrauterine contraceptive device
86053994	Intrauterine contraceptive device
87355979	Intrauterine contraceptive device
87911994	Intrauterine contraceptive device
89797994	Intrauterine contraceptive device
89798994	Intrauterine contraceptive device
90662994	Intrauterine contraceptive device
91073994	Intrauterine contraceptive device
91074994	Intrauterine contraceptive device
91271994	Intrauterine contraceptive device
91324998	Levonorgestrel 20micrograms/24hours intrauterine device
91325998	Levonorgestrel 20micrograms/24hours intrauterine device
92849979	Levonorgestrel 20micrograms/24hours intrauterine device
92851979	Levonorgestrel 20micrograms/24hours intrauterine device
95678994	Intrauterine contraceptive device
97021994	Intrauterine contraceptive device

97339992	Intrauterine contraceptive device
97916994	Intrauterine contraceptive device
97917994	Intrauterine contraceptive device
98212994	Intrauterine contraceptive device
99235994	Intrauterine contraceptive device
99880994	Intrauterine contraceptive device
81886998	Etonogestrel 68mg implant
90908998	Etonogestrel 68mg implant
90909998	Etonogestrel 68mg implant
92888998	Levonorgestrel 38mg implant
98222998	Levonorgestrel 228mg implant
84519978	Medroxyprogesterone 104mg/0.65ml suspension for injection pre-filled disposable devices
84520978	Medroxyprogesterone 104mg/0.65ml suspension for injection pre-filled disposable devices
85241998	Medroxyprogesterone 150mg/1ml suspension for injection pre-filled syringes
85242998	Medroxyprogesterone 150mg/1ml suspension for injection pre-filled syringes
92842979	Medroxyprogesterone 150mg/1ml suspension for injection pre-filled syringes
92843979	Medroxyprogesterone 150mg/1ml suspension for injection pre-filled syringes
92844979	Medroxyprogesterone 150mg/1ml suspension for injection pre-filled syringes
92846979	Medroxyprogesterone 150mg/1ml suspension for injection pre-filled syringes
92847979	Medroxyprogesterone 150mg/1ml suspension for injection pre-filled syringes
95700998	Norethisterone 200mg/1ml solution for injection ampoules
97454998	Norethisterone 200mg/1ml solution for injection ampoules
97920998	Medroxyprogesterone 150mg/1ml suspension for injection pre-filled syringes

9. Long-acting reversible contraception Read codes

Read code	Description
6151.00	IUD fitted
6153.00	IUD re-fitted
61A2.00	"Morning after" IUD fitted
61A2.11	Post-coital IUD fitted
7E09.12	Intrauterine device procedure
7E09000	Introduction of intrauterine contraceptive device
7E09011	Fitting of intrauterine contraceptive device
7E09100	Replacement of intrauterine contraceptive device
7E09111	Change of intrauterine contraceptive device
7E09400	Introduction of Mirena coil
7E09600	Replacement of intrauterine system
7E09700	Insertion of intrauterine system
ZV25100	[V]Intrauterine contraceptive device insertion
ZV25112	[V]Intrauterine contraceptive device insertion
ZV25113	[V]Intrauterine contraceptive device insertion
ZV25412	[V]Reinsertion of coil
ZV25415	[V]Reinsertion of intrauterine contraceptive device
ZV2541A	[V]Reinsertion of intrauterine contraceptive device
ZV25D00	[V]Reinsertion of intrauterine contraceptive device
ZV25D11	[V]Reinsertion of coil

7G2AG00	Insertion of Implanon
7G2AJ00	Insertion of etonogestrel radiopaque contraceptive implant
61KA.00	Insertion of subcutaneous contraceptive
61KC.00	Insert subcutaneous contraceptive implnt othr healthcre prov
7G2AB00	Insertion of subcutaneous contraceptive
7G2AH00	Reinsertion of subcutaneous contraceptive
61B..00	Depot contraceptive
61B..11	Depot contraception
61B1.00	Depot contraceptive given
61B1.11	Depo-provera injection given
61B2.00	Depot contraceptive repeated
61B3.00	Depot contraceptive-no problem
61BZ.00	Depot contraceptive NOS

10. Hormone-replacement therapy

Drug code	Generic name
54611979	Estradiol 500micrograms / Dydrogesterone 2.5mg tablets
60462979	Estradiol 50micrograms/24hours / Levonorgestrel 7micrograms/24hours transdermal patches
60489979	Estradiol 25micrograms/24hours transdermal patches
60490979	Estradiol 25micrograms/24hours transdermal patches
78588978	Estradiol 50micrograms/24hours transdermal patches
82739998	Estradiol 1mg gel sachets
82740998	Estradiol 1mg gel sachets
82741998	Estradiol 500microgram gel sachets
82742998	Estradiol 500microgram gel sachets
83058998	Generic hormonin tablets
83429998	Estradiol 100micrograms/24hours transdermal patches
83430998	Estradiol 75micrograms/24hours transdermal patches
83431998	Estradiol 25micrograms/24hours transdermal patches
83432998	Estradiol 50micrograms/24hours transdermal patches
84780998	Conjugated oestrogens 300microgram tablets
84781998	Conjugated oestrogens 300microgram tablets
84862998	Estradiol 0.06% gel (750microgram per actuation)
85771979	Ethinylestradiol 33.9micrograms/24hours / Norelgestromin 203micrograms/24hours transdermal patches
85772979	Ethinylestradiol 33.9micrograms/24hours / Norelgestromin 203micrograms/24hours transdermal patches
85962998	Estradiol 100micrograms/24hours transdermal patches
85963998	Estradiol 100micrograms/24hours transdermal patches
85964998	Estradiol 75micrograms/24hours transdermal patches
85965998	Estradiol 50micrograms/24hours transdermal patches
85966998	Estradiol 50micrograms/24hours transdermal patches
85967998	Estradiol 25micrograms/24hours transdermal patches
85973998	Estradiol 100micrograms/24hours transdermal patches
85974998	Estradiol 75micrograms/24hours transdermal patches
85975998	Estradiol 50micrograms/24hours transdermal patches
85976998	Estradiol 25micrograms/24hours transdermal patches

86050998	Generic Clinorette tablets
86058998	Estradiol 2mg tablets
86546979	Estradiol 1mg gel sachets
86831998	Estradiol 1mg / Drospirenone 2mg tablets
86832998	Estradiol 1mg / Drospirenone 2mg tablets
87042998	Estradiol 100micrograms/24hours transdermal patches
87043998	Estradiol 75micrograms/24hours transdermal patches
87044998	Estradiol 50micrograms/24hours transdermal patches
87045998	Estradiol 37.5micrograms/24hours transdermal patches
87046998	Estradiol 25micrograms/24hours transdermal patches
87047998	Estradiol 100micrograms/24hours transdermal patches
87048998	Estradiol 75micrograms/24hours transdermal patches
87049998	Estradiol 50micrograms/24hours transdermal patches
87050998	Estradiol 37.5micrograms/24hours transdermal patches
87051998	Estradiol 25micrograms/24hours transdermal patches
87076979	Generic Femoston 2/10mg tablets
87082979	Generic Evorel Sequi transdermal patches
87549998	Conjugated oestrogens 300microgram / Medroxyprogesterone 1.5mg modified-release tablets
87550998	Conjugated oestrogens 300microgram / Medroxyprogesterone 1.5mg modified-release tablets
87759998	Ethinylestradiol 2microgram tablets
87898979	Generic climagest 1mg tablets
87901979	Generic climagest 1mg tablets
87953998	Conjugated oestrogens equine with medroxyprogesterone acetate 625micrograms with 10mg tablets
88207998	Estradiol 1mg / Dydrogesterone 5mg tablets
88320998	Estradiol 2mg / Norethisterone acetate 1mg tablets
88327998	Estradiol 100micrograms/24hours transdermal patches
88329998	Estradiol 75micrograms/24hours transdermal patches
88331997	Estradiol 100micrograms/24hours transdermal patches
88331998	Estradiol 75micrograms/24hours transdermal patches
88561998	Phyto progesterone cream
88634979	Estradiol 1mg / Dydrogesterone 5mg tablets
88635979	Estradiol 1mg / Dydrogesterone 5mg tablets
88638979	Estradiol 1mg / Dydrogesterone 5mg tablets
88826998	Estradiol 80micrograms/24hours transdermal patches
88828998	Estradiol 40micrograms/24hours transdermal patches
88835998	Estradiol 50micrograms/24hours transdermal patches
88887997	Generic Evorel Sequi transdermal patches
88887998	Generic Evorel Sequi transdermal patches
88889998	Estradiol 50micrograms/24hours / Norethisterone 170micrograms/24hours transdermal patches
88912998	Estradiol valerate 2mg / norethisterone 700microgram tablets
88915998	Estradiol 1mg gel sachets
88935998	Estradiol hemihydrate 150mcg nasal spray
88937998	Estradiol 150micrograms/dose nasal spray
89082996	Estradiol 100micrograms/24hours transdermal patches
89082997	Estradiol 50micrograms/24hours transdermal patches
89082998	Estradiol 25micrograms/24hours transdermal patches

89171979	Conjugated estrogens & medroxyprogesterone 0.625mg+5mg tablets
89173979	Conjugated estrogens & medroxyprogesterone 0.625mg+5mg tablets
89176979	Conjugated estrogens & medroxyprogesterone 0.625mg+5mg tablets
89209996	Estradiol 75micrograms/24hours transdermal patches
89209997	Estradiol 50micrograms/24hours transdermal patches
89209998	Estradiol 25mcg transdermal patches
89212998	Estradiol and (estradiol with levonorgestrel) 80mcg/24hrs with (50mcg+20mcg/24hr) twice weekly patch
89216998	Generic nuvelle ts transdermal patches
89253998	Phyto progesterone cream
89295998	Ethinylestradiol 33.9micrograms/24hours / Norelgestromin 203micrograms/24hours transdermal patches
89321998	Generic FemSeven Sequi transdermal patches
89359998	Estradiol with dydrogesterone 1mg +10mg tablets
89399998	Estradiol 1mg / Norethisterone acetate 500microgram tablets
89469998	Generic Novofem tablets
89500998	Estradiol 50mcg/24hours vaginal ring
89627998	Estradiol 75micrograms/24hours transdermal patches
89629998	Estradiol 75micrograms/24hours transdermal patches
89684979	Conjugated oestrogens 625microgram tablets and norgestrel 150microgram tablets
89685979	Conjugated oestrogens 625microgram tablets and norgestrel 150microgram tablets
89722979	Estradiol 50micrograms/24hours / Norethisterone 170micrograms/24hours transdermal patches
89723979	Estradiol 50micrograms/24hours / Norethisterone 170micrograms/24hours transdermal patches
89725979	Estradiol 50micrograms/24hours / Norethisterone 170micrograms/24hours transdermal patches
89803998	Estradiol 1mg / dydrogesterone 5mg tablets
89869998	Phyto progesterone 1.5% cream
89901998	Progesterone 3% cream
89907998	Phyto progesterone 3% cream
89953998	Estradiol 2mg tablets
90083998	Generic Evorel Sequi transdermal patches
90241996	Estradiol 100micrograms/24hours transdermal patches
90241997	Estradiol 50micrograms/24hours transdermal patches
90241998	Estradiol 25micrograms/24hours transdermal patches
90247996	Estradiol 100micrograms/24hours transdermal patches
90247997	Estradiol 50micrograms/24hours transdermal patches
90247998	Estradiol 25micrograms/24hours transdermal patches
90523998	Estradiol valerate & norethisterone 2mg+0.7mg tablets
90617998	Estradiol valerate 2mg / Medroxyprogesterone 5mg tablets
90618996	Estradiol valerate 2mg / Medroxyprogesterone 5mg tablets
90618997	Estradiol valerate 1mg / Medroxyprogesterone 2.5mg tablets
90618998	Estradiol valerate 1mg / Medroxyprogesterone 5mg tablets
90620998	Estradiol 40micrograms/24hours transdermal patches and dydrogesterone 10mg tablets
90645998	Estradiol 50micrograms/24hours / Levonorgestrel 7micrograms/24hours transdermal patches

90646998	Estradiol 50micrograms/24hours / levonorgestrel 7micrograms/24hours transdermal patches
90770998	Piperazine oestrone sulphate 1.5mg with medroxyprogesterone 10mg tablet
90771998	Piperazine oestrone sulphate 1.5mg with medroxyprogesterone 10mg tablet
90813998	Estradiol acetate 1.25mg vaginal ring
90819997	Estradiol 100micrograms/24hours transdermal patches
90819998	Estradiol 50micrograms/24hours transdermal patches
90834996	Estradiol 25micrograms/24hours transdermal patches
90834997	Estradiol 100micrograms/24hours transdermal patches
90834998	Estradiol 75micrograms/24hours transdermal patches
90835996	Estradiol 100micrograms/24hours transdermal patches
90835997	Estradiol 50micrograms/24hours transdermal patches
90835998	Estradiol 50micrograms/24hours transdermal patches
90873997	Generic Elleste Duet 1mg tablets
90873998	Generic Elleste Duet 2mg tablets
90875997	Estradiol 2mg tablets
90875998	Estradiol 1mg tablets
90894998	Estradiol 50micrograms/24hours transdermal patches
91052998	Estradiol 80micrograms/24hours transdermal patches and dydrogesterone 10mg tablets
91054998	Estradiol 40micrograms/24hours transdermal patches
91086998	Estradiol valerate 2mg / Norethisterone 1mg tablets
91090996	Estradiol 75micrograms/24hours transdermal patches
91090997	Estradiol 50micrograms/24hours transdermal patches
91090998	Estradiol 37.5micrograms/24hours transdermal patches
91096998	Conjugat oestrogen equi and (conjugat oestrogen equi with medroxyprogesterone acetate 625 micrograms with (625 microgram
91097998	Generic premique cycle tablets
91113998	Conjugated oestrogens 625microgram / medroxyprogesterone 5mg tablets
91114998	Conjugated estrogens & medroxyprogesterone 0.625mg+5mg tablets
91307997	Estradiol 40micrograms/24hours transdermal patches and dydrogesterone 10mg tablets
91307998	Estradiol 80micrograms/24hours transdermal patches and dydrogesterone 10mg tablets
91328998	Generic adgyn combi tablets
91350996	Estradiol valerate 1mg / Medroxyprogesterone 2.5mg tablets
91350997	Estradiol valerate 1mg / Medroxyprogesterone 5mg tablets
91350998	Estradiol valerate 1mg / Medroxyprogesterone 2.5mg tablets
91351998	Generic Tridestra tablets
91388996	Estradiol and (estradiol with dydrogesterone) 2mg with (2mg with 20mg) tablets
91388997	Estradiol and (estradiol with dydrogesterone) 2mg with (2mg with 10 mg) tablets
91388998	Estradiol and (estradiol with dydrogesterone) 1mg with (1mg with 10mg) tablets
91389996	Generic femoston 2/20mg tablets
91389997	Generic Femoston 2/10mg tablets
91389998	Generic Femoston 1/10mg tablets
91399997	Estradiol 1mg gel sachets
91399998	Estradiol 0.06% gel (750microgram per actuation)
91400998	Estradiol 1.25g/dose gel
91412996	Estradiol 1mg / Norethisterone acetate 500microgram tablets
91412997	Estradiol with norethisterone acetate (continuous combined) 2mg with 0.7mg

	tablets
91412998	Estradiol valerate 2mg / Norethisterone 1mg tablets
91423998	Estradiol 2mg / Norethisterone acetate 1mg tablets
91457998	Estradiol 80micrograms/24hours transdermal patches
91469998	Estradiol and (estradiol with levonorgestrel) 50mcg/24hrs with (50mcg+10mcg/24hrs) once weekly patch
91479998	Generic Tridestra tablets
91546998	Estradiol valerate 2mg / Norethisterone 1mg tablets
91560998	Progesterone 1.5% cream
91620996	Estradiol 75micrograms/24hours transdermal patches
91620997	Estradiol 50micrograms/24hours transdermal patches
91620998	Estradiol 37.5micrograms/24hours transdermal patches
91680998	Estradiol with norethisterone acetate 50mcg/24hours(4mg/unit) with 1mg patch with tablet
91859998	Estradiol valerate 1mg tablets
91862998	Estradiol 2mg / Norethisterone acetate 1mg tablets
91864998	Generic nuvelle tablets
91865998	Estradiol valerate 2mg tablets
91871998	Estradiol valerate with norgestrel 2mg+500micrograms tablets
91878998	Ethinylestradiol 33.9micrograms/24hours / Norelgestromin 203micrograms/24hours transdermal patches
92065998	Estradiol 100micrograms/24hours transdermal patches
92171998	Estradiol 1mg / Dydrogesterone 5mg tablets
92221998	Estradiol 25micrograms/24hr once weekly patch
92251998	Estradiol with (estradiol with norethisterone acetate) 1mg with (1mg with 1mg) tablets
92366998	Estradiol 100micrograms/24hours transdermal patches
92371998	Estradiol 50micrograms/24hours transdermal patches
92440998	Estradiol 2mg / Norethisterone acetate 1mg tablets
92585998	Estradiol with norethisterone 0mcg/24hours(3.2mg/unit) with 1mg patch with tablet
92586998	Estradiol 50micrograms/24hours transdermal patches and norethisterone 1mg tablets
92962996	Estradiol 40micrograms/24hours transdermal patches
92962997	Estradiol 80micrograms/24hours transdermal patches
92962998	Estradiol 100micrograms/24hours transdermal patches
93073996	Estradiol 75micrograms/24hours transdermal patches
93073997	Estradiol 25micrograms/24hours transdermal patches
93073998	Estradiol 50micrograms/24hours transdermal patches
93164979	Estradiol valerate & norethisterone 2mg+0.7mg tablets
93165979	Estradiol valerate & norethisterone 2mg+0.7mg tablets
93169979	Estradiol 2mg / Norethisterone acetate 1mg tablets
93174979	Estradiol 2mg / Norethisterone acetate 1mg tablets
93189992	Estradiol 50micrograms/24hours transdermal patches
93191979	Estradiol 0.06% gel (750microgram per actuation)
93192979	Estradiol 0.06% gel (750microgram per actuation)
93193979	Estradiol 0.06% gel (750microgram per actuation)
93194979	Estradiol 0.06% gel (750microgram per actuation)
93195979	Estradiol 0.06% gel (750microgram per actuation)
93197979	Tibolone 2.5mg tablets

93201979	Tibolone 2.5mg tablets
93204979	Tibolone 2.5mg tablets
93211979	Conjugated oestrogens 625microgram tablets
93251979	Estradiol 100micrograms/24hours transdermal patches
93254979	Estradiol 100micrograms/24hours transdermal patches
93260979	Estradiol 100micrograms/24hours transdermal patches
93262979	Estradiol 100micrograms/24hours transdermal patches
93267979	Estradiol 50micrograms/24hours transdermal patches
93269979	Estradiol 50micrograms/24hours transdermal patches
93276979	Estradiol 50micrograms/24hours transdermal patches
93278979	Estradiol 50micrograms/24hours transdermal patches
93281979	Estradiol 50micrograms/24hours transdermal patches
93283979	Estradiol 50micrograms/24hours transdermal patches
93284979	Estradiol 50micrograms/24hours transdermal patches
93285979	Estradiol 50micrograms/24hours transdermal patches
93287979	Estradiol 50micrograms/24hours transdermal patches
93288979	Estradiol 50micrograms/24hours transdermal patches
93293979	Estradiol 25micrograms/24hours transdermal patches
93296979	Estradiol 25micrograms/24hours transdermal patches
93303979	Estradiol 25micrograms/24hours transdermal patches
93308979	Estradiol 75micrograms/24hours transdermal patches
93311979	Estradiol 75micrograms/24hours transdermal patches
93315998	Tibolone 2.5mg tablets
93319998	Tibolone 2.5mg tablets
93321979	Estradiol valerate 2mg tablets
93325979	Estradiol valerate 2mg tablets
93336992	Ethinyloestradiol 5 mg tab
93341979	Estradiol valerate 1mg tablets
93352979	Estradiol 37.5micrograms/24hours transdermal patches
93354979	Estradiol 80micrograms/24hours transdermal patches
93387992	Ethinyloestradiol 2 mcg tab
93461992	Oestradiol 17b
93578998	Ethinylestradiol 1mg tablets
93696997	Estradiol valerate 2mg tablets
93696998	Estradiol valerate 1mg tablets
93764992	Conjugated oestrogens / norgestrel 1.25 mg tab
94156992	Ethinyloestradiol 15 mcg tab
94161997	Estradiol and (estradiol with norethisterone) and (estradiol) triphasic forte 4mg with (4mg with 1mg) with (1mg) tablets
94161998	Generic Trisequens tablets
94162998	Generic Cyclo-Progynova 2mg tablets
94252992	Conjugated oestrogens 625/norgestrel 500 mcg tab
94309992	Prempak 1.25mg mg tab
94361992	Tace 12 mg cap
94458992	Ethinyloestradiol 25 mcg tab
94472997	Conjugated oestrogens 1.25mg tablets and norgestrel 150microgram tablets
94472998	Conjugated oestrogens 625microgram tablets and norgestrel 150microgram tablets
94516996	Estradiol 100micrograms/24hours transdermal patches
94516997	Estradiol 50micrograms/24hours transdermal patches

94516998	Estradiol 25micrograms/24hours transdermal patches
94517998	Estradiol 50micrograms/24hours transdermal patches and norethisterone acetate 1mg tablets
94518996	Estradiol 25micrograms/24hours transdermal patches
94518997	Estradiol 100micrograms/24hours transdermal patches
94518998	Estradiol 25micrograms/24hours transdermal patches
94519996	Estradiol 75micrograms/24hours transdermal patches
94519997	Estradiol 25micrograms/24hours transdermal patches
94519998	Estradiol 50micrograms/24hours transdermal patches
94737997	Estradiol valerate 2mg tablets
94737998	Estradiol valerate 1mg tablets
94918998	Ethinylestradiol 33.9micrograms/24hours / Norelgestromin 203micrograms/24hours transdermal patches
94971998	Estropipate 1.5mg tablets
94989992	Ethinyloestradiol 30 mcg tab
94990992	Ethinyloestradiol 100 mcg tab
95339998	Quinestradol 250mcg tablets
95351992	Estradiol 50mg implant
95363992	Estriol 250mcg tablets
95603998	Generic nuvelle tablets
95657997	Estradiol valerate and (estradiol valerate with levonorgestrel) 1mg with (1mg with 250micrograms) tablets
95657998	Estradiol valerate and (estradiol valerate with levonorgestrel) 2mg with (2mg with 75micrograms) tablets
95698997	Norgestrel and conjugated oestrogens (equine) 150micrograms + 1.25mg tablet
95698998	Norgestrel and conjugated oestrogens (equine) 150micrograms + 625micrograms tablet
96371992	Oestradiol .01 mg tab
96392997	Dienestrol 5mg tablets
96392998	Dienestrol 1mg tablets
96609996	Conjugated oestrogens 2.5mg tablets
96609997	Conjugated oestrogens 1.25mg tablets
96609998	Conjugated oestrogens 625microgram tablets
96744997	Estriol 1mg tablets
96744998	Estriol 250micrograms tablets
96745998	Estradiol with estrone and estriol tablets
96746992	Premarin 1.25mg/norgestrel 0.15mg mg tab
96746998	Estradiol with estrone and estriol tablets
96747996	Estradiol 40micrograms/24hours transdermal patches
96747997	Estradiol 1mg tablets
96747998	Estradiol 2mg tablets
96748997	Estradio; 5m/ml injection
96748998	Estradiol 1mg/ml injection
96892992	Estradiol 50micrograms/24hours transdermal patches
97387992	Ethinyloestradiol 20 mcg pes
97397992	Ethinyloestradiol 5 mcg cap
97404992	Ethisterone 5 mg tab
97457997	Estradiol valerate 2mg tablets
97457998	Estradiol valerate 1mg tablets
97458997	Generic Cyclo-Progynova 2mg tablets

97458998	Generic cyclo-progynova 1mg tablets
97482997	Generic trisequens forte tablets
97482998	Generic Trisequens tablets
97625997	Estradiol valerate (2mg) with norethisterone (1 mg) tablets
97625998	Estradiol valerate and (estradiol valerate with norethisterone) 1mg with (1mg with 1mg) tablets
97732998	Generic estracombi tts transdermal patches
97759996	Estradiol 50micrograms/24hours / Norethisterone 170micrograms/24hours transdermal patches
97759998	Estradiol with (estradiol with norethisterone acetate) 50mcg/24 hr with (50mcg+250mcg/24 hr) twice weekly patch
97762997	Estradiol 1mg tablets
97762998	Estradiol 2mg tablets
97765997	Estradiol & norethisterone acetate 2mg+1mg tablets
97765998	Generic climagest 1mg tablets
97826992	Estradiol 1mg tablets
97947992	Premarin 0.625mg/norgestrel 0.15mg mg tab
97993996	Ethinylestradiol 50microgram tablets
97993997	Ethinylestradiol 20micrograms tablet
97993998	Ethinylestradiol 10microgram tablets
98468989	Estradiol 50mg implant
98468990	Estradiol 25mg implant
98728998	Ethinylestradiol with methyltestosterone 4.4micrograms + 3.6mg tablet
98839998	Conjugated oestrogens 1.25mg tablets and norgestrel 150microgram tablets
98840998	Conjugated oestrogens 1.25mg tablets and norgestrel 150microgram tablets
98892998	Conjugated oestrogens 625microgram tablets and norgestrel 150microgram tablets
98897998	Mestranol with norethisterone tablet
98911996	Estradiol 100mg implant
98911997	Estradiol 50mg implant
98911998	Estradiol 25mg implant
99219998	Conjugated oestrogens 625microgram tablets and norgestrel 150microgram tablets
99220996	Conjugated estrogens 2.5mg tablets
99220997	Conjugated oestrogens 1.25mg tablets
99220998	Conjugated oestrogens 625microgram tablets
99295997	Estriol 1mg tablets
99295998	Estriol 250mcg tablets
99571998	Estropipate 1.5mg tablets
99602989	Ethinylestradiol 50microgram tablets
99602990	Ethinylestradiol 10microgram tablets

11. Hysterectomy, bilateral salpingo-oophorectomy & sterilisation

Read code	Description
1599.00	H/O: hysterectomy
159A.11	H/O: sterilisation - female
159B.00	H/O: bilateral oophorectomy
15A9.11	H/O: hysterotomy

61H..00	Contraception: female sterilis
685H.00	No smear - benign hysterectomy
685H.11	No smear - hysterectomy
7E04.00	Abdominal excision of uterus
7E04.11	Abdominal hysterectomy
7E04.12	Wertheim hysterectomy
7E04000	Abdominal hysterocolpectomy and excision periuterine tissue
7E04100	Abdominal hysterectomy & excision of periuterine tissue NEC
7E04200	Abdominal hysterocolpectomy NEC
7E04300	Total abdominal hysterectomy NEC
7E04311	Bonney abdominal hysterectomy
7E04312	Hysterectomy NEC
7E04400	Subtotal abdominal hysterectomy
7E04500	Abdominal hysterectomy and bilateral salpingoophorectomy
7E04511	Abdominal hysterectomy & bilateral salpingoophorectomy (BSO)
7E04512	TAH - total abdom hysterectomy & bilateral salpingoophorect
7E04600	Radical hysterectomy
7E04700	Abdominal hysterectomy and right salpingoopherectomy
7E04711	Abdominal hysterectomy and left salpingoopherectomy
7E04800	Abdominal hysterectomy and left salpingoophorectomy
7E04900	TAH - Tot abdom hysterectomy and BSO - bilat salpingophorect
7E04A00	Abdominal hysterectomy with conservation of ovaries
7E04B00	Lapar total abdominal hysterect bilat salpingo-oophorectomy
7E04C00	Laparoscopic hysterectomy
7E04E00	Laparoscopic subtotal hysterectomy
7E04F00	Subtotal abdominal hysterectomy with conservation of ovaries
7E04G00	Total abdominal hysterectomy with conservation of ovaries
7E04H00	Subtotl abdominal hysterectomy & bilat salpingo-oophorectomy
7E04J00	Subtotl abdominal hysterectomy & right salpingo-oophorectomy
7E04K00	Subtotal abdominal hysterectomy & left salpingo-oophorectomy
7E04N00	Radical hysterectomy with conservation of ovaries
7E04P00	Radical hysterectomy with bilateral salpingo-oophorectomy
7E04y00	Other specified abdominal excision of uterus
7E04z00	Abdominal excision of uterus NOS
7E05.00	Vaginal excision of uterus
7E05.11	Schauta radical vaginal hysterectomy
7E05.12	Vaginal hysterectomy
7E05000	Vaginal hysterocolpectomy and excision of periuterine tissue
7E05100	Vaginal hysterectomy and excision of periuterine tissue NEC
7E05200	Vaginal hysterocolpectomy NEC
7E05300	Vaginal hysterectomy NEC
7E05311	Heaney vaginal hysterectomy
7E05400	Laparoscopic vaginal hysterectomy
7E05500	Vaginal hysterectomy with conservation of ovaries
7E05600	Lap assist vag hysterectomy with bilat salpingo-oophorectomy
7E05700	Vaginal hysterectomy and right salpingo-oophorectomy
7E05800	Vaginal hysterectomy and left salpingo-oophorectomy
7E05y00	Other specified vaginal excision of uterus
7E05y11	Ward vaginal hysterectomy
7E05z00	Vaginal excision of uterus NOS

7E10000	Bilateral salpingoophorectomy
7E10100	Bilateral salpingectomy NEC
7E10200	Bilateral oophorectomy NEC
7E11100	Salpingoophorectomy remaining solitary fallop tube and ovary
7E11300	Salpingectomy of remaining solitary fallopian tube NEC
7E11500	Oophorectomy of remaining solitary ovary NEC
7E15.00	Open bilateral occlusion of fallopian tubes
7E15.11	Open bilateral female sterilisation
7E15000	Open bilateral ligation of fallopian tubes
7E15011	Pomeroy open bilateral ligation of fallopian tubes
7E15100	Open bilateral clipping of fallopian tubes
7E15111	Open bilateral ringing of fallopian tubes
7E15y00	Other specified open bilateral occlusion of fallopian tubes
7E15z00	Open bilateral occlusion of fallopian tubes NOS
7E16.11	Other open female sterilisation
7E16000	Open ligation of remaining solitary fallopian tube
7E16200	Open clipping of remaining solitary fallopian tube
7E16211	Open clipping of residual solitary fallopian tube
7E16212	Open ringing of remaining solitary fallopian tube
7E1C.00	Endoscopic bilateral occlusion of fallopian tubes
7E1C.11	Endoscopic bilateral female sterilisation
7E1C.12	Laparoscopic bilateral female sterilisation
7E1C000	Endoscopic bilateral cauterisation of fallopian tubes
7E1C100	Endoscopic bilateral clipping of fallopian tubes
7E1C200	Endoscopic bilateral ringing of fallopian tubes
7E1C300	Endoscopic bilateral placement of intrafallopian implants
7E1Cy00	Endoscopic bilateral occlusion of fallopian tubes OS
7E1Cz00	Endoscopic bilateral occlusion of fallopian tubes NOS
7E1D.12	Other endoscopic female sterilisation
7E1D.13	Other laparoscopic female sterilisation
7E1D000	Endoscopic occlusion of remaining solitary fallopian tube
7E1D300	Endo place intrafallop implant remain solitary fallop tube
7F1A000	Caesarean hysterectomy
9O8W.00	Cervical smear to continue post hysterectomy
K515.00	Post hysterectomy vaginal vault prolapse
L398500	Delivery by caesarean hysterectomy
ZV25200	[V]Sterilisation
