# Supplementary material for: Time trends in contraceptive prescribing in UK primary care 2000–2018: a repeated cross-sectional study
Source: BMJ Sex Reprod Health. 2021 Nov 15;48(3):193–8. doi: 10.1136/bmjsrh-2021-201260 (PMC9279840; doi:10.1136/bmjsrh-2021-201260)

SUPPLEMENTARY FIGURES:

Fig.1 Temporal trends in combined hormonal contraception prescribing by country over the period 2000-2018

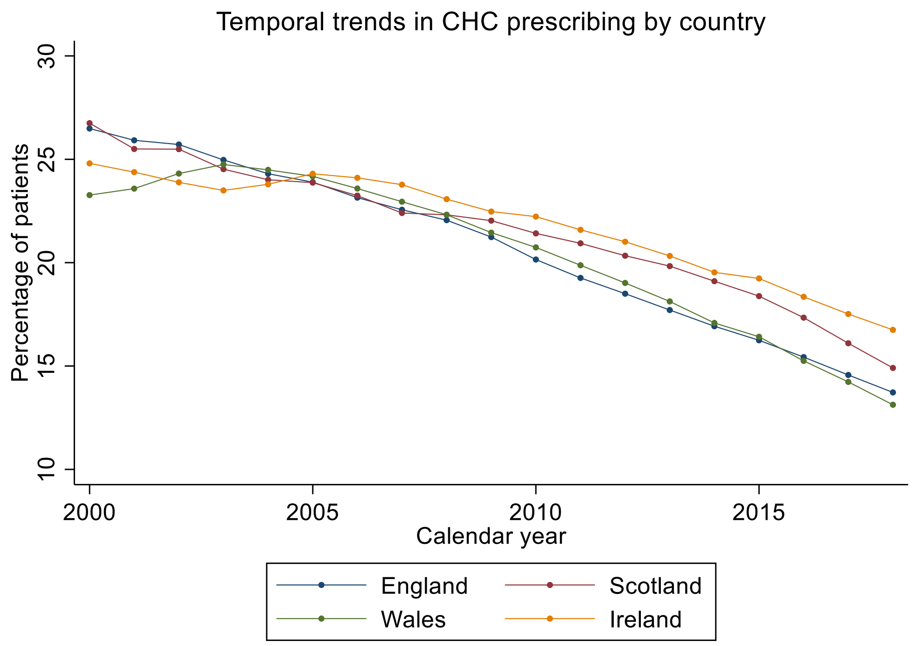

Fig.2 Temporal trends in combined hormonal contraception prescribing by social deprivation quintile over the period 2000-2018

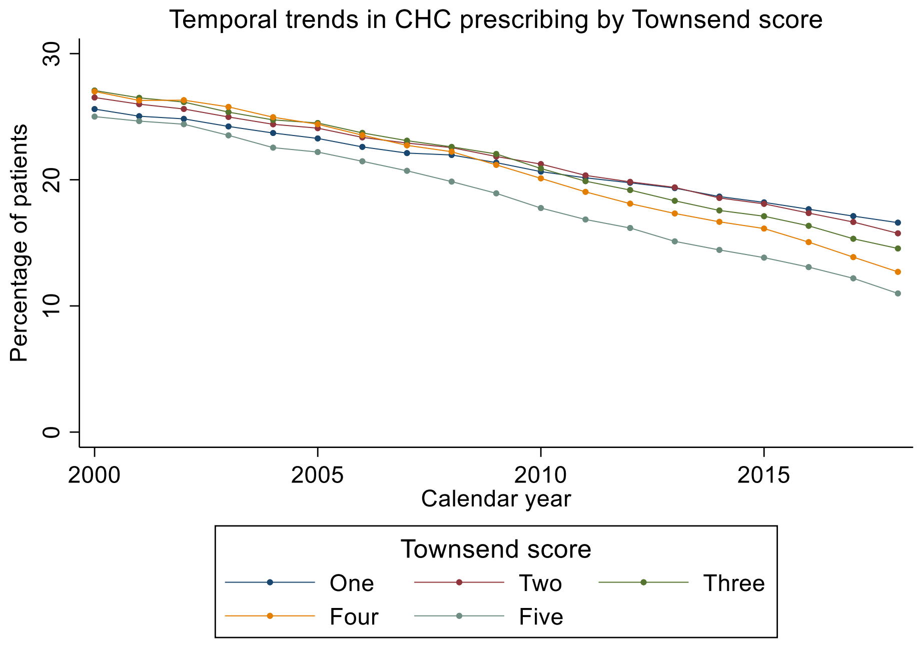

Fig.3 Temporal trends in combined hormonal contraception prescribing by age group over the period 2000-2018

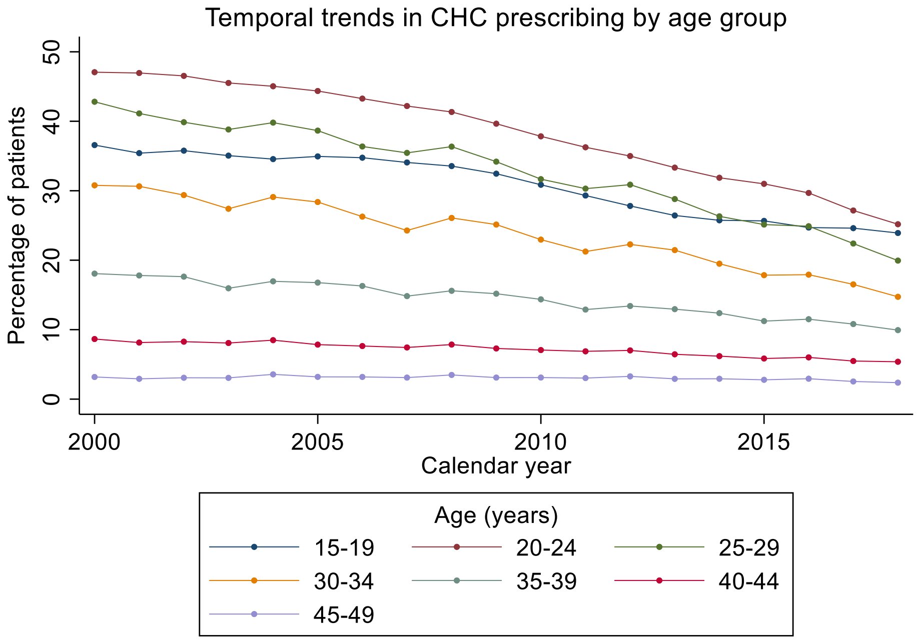

Fig.4 Temporal trends in progestogen-only pill prescribing by country over the period 2000-2018

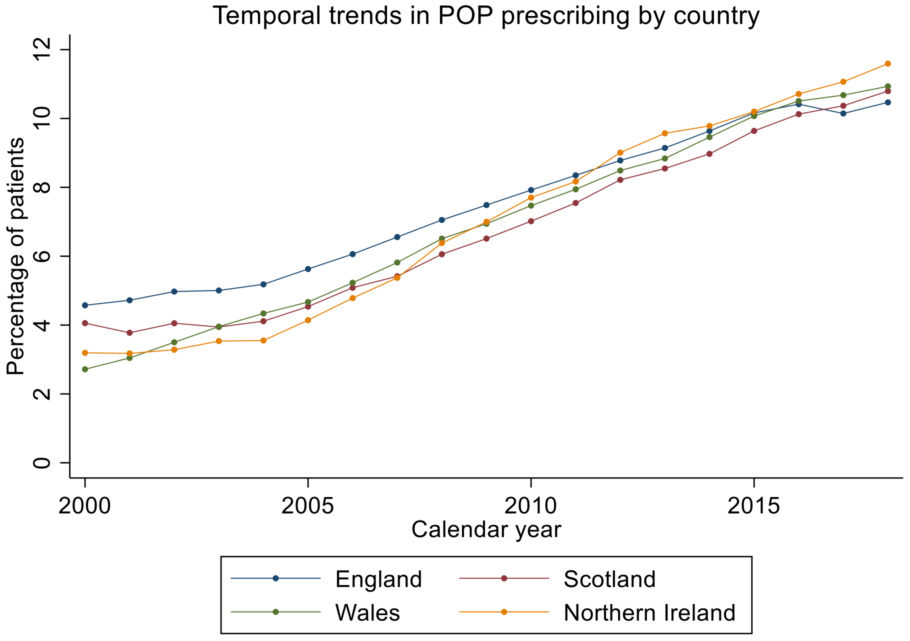

Fig.5 Temporal trends in progestogen-only pill prescribing by social deprivation quintile over the period 2000-2018

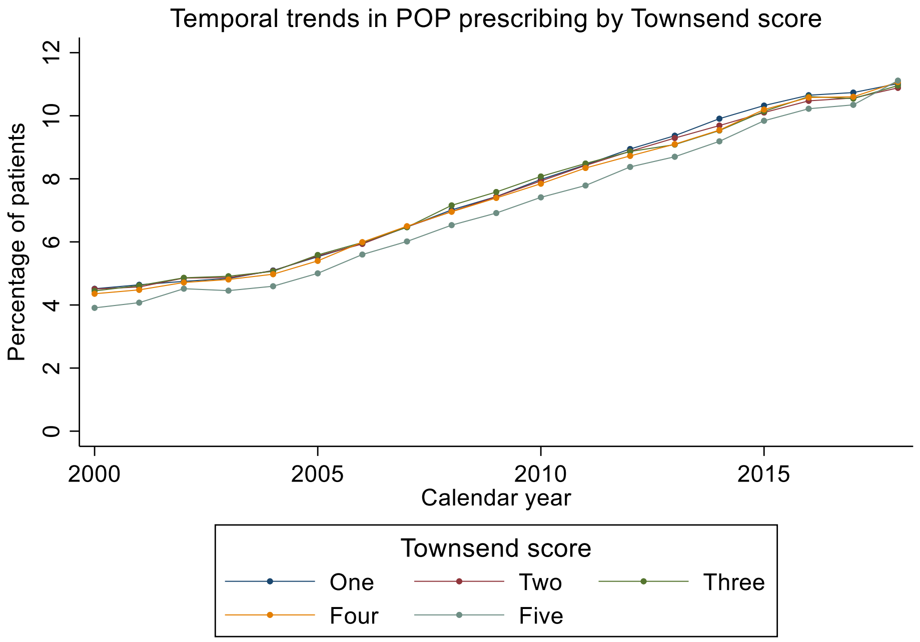

Fig.6 Temporal trends in progestogen-only pill prescribing by age group over the period 2000-2018

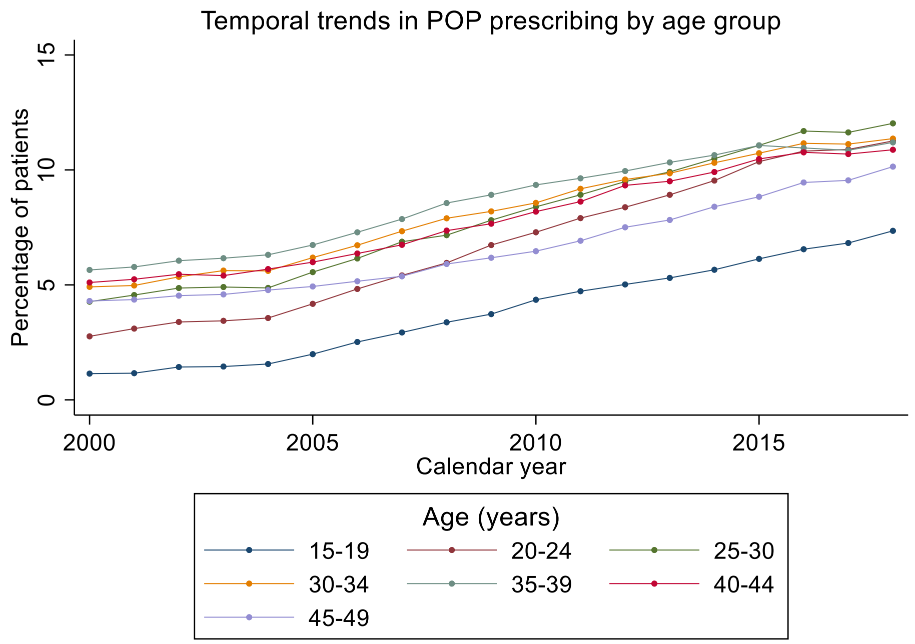

Fig.7 Temporal trends in long-acting reversible contraceptive prescribing by country over the period 2000-2018

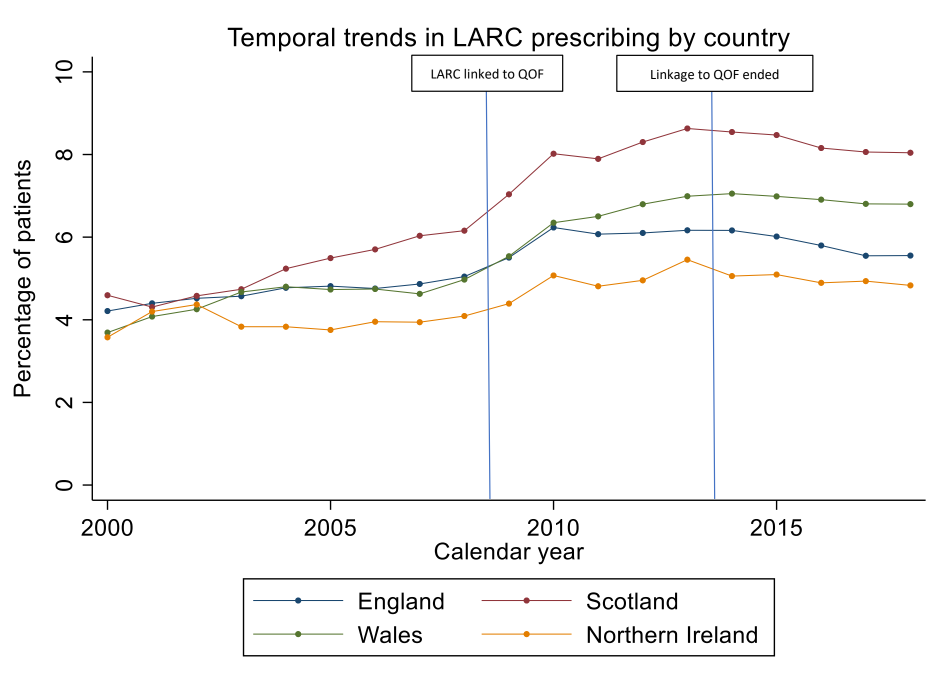

Fig.8 Temporal trends in long-acting reversible contraceptive prescribing by social deprivation quintile over the period 2000-2018

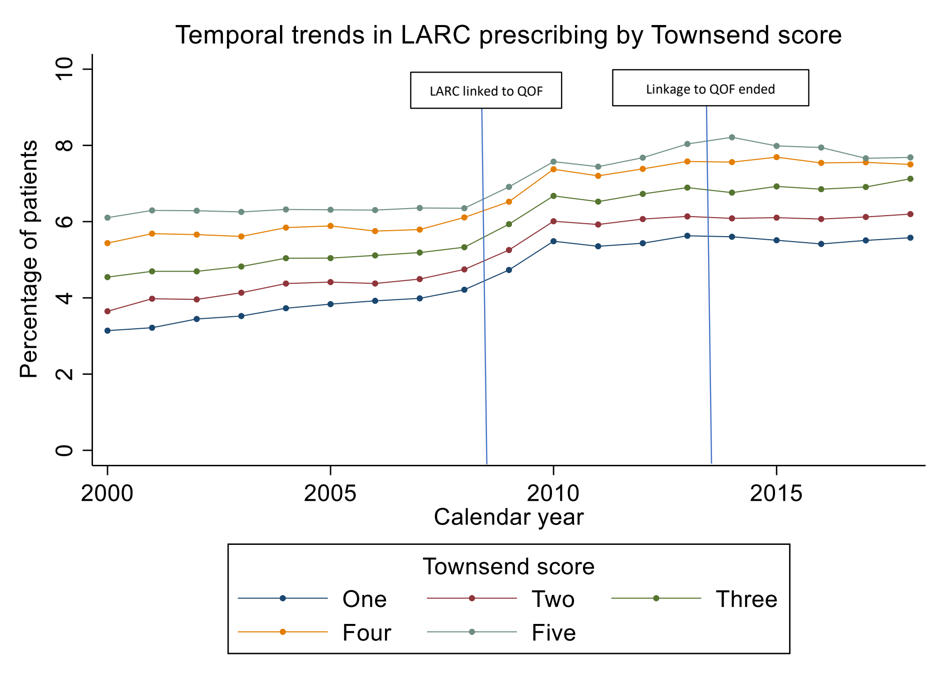

Fig.9 Temporal trends in long-acting reversible contraceptive prescribing by age group over the period 2000-2018

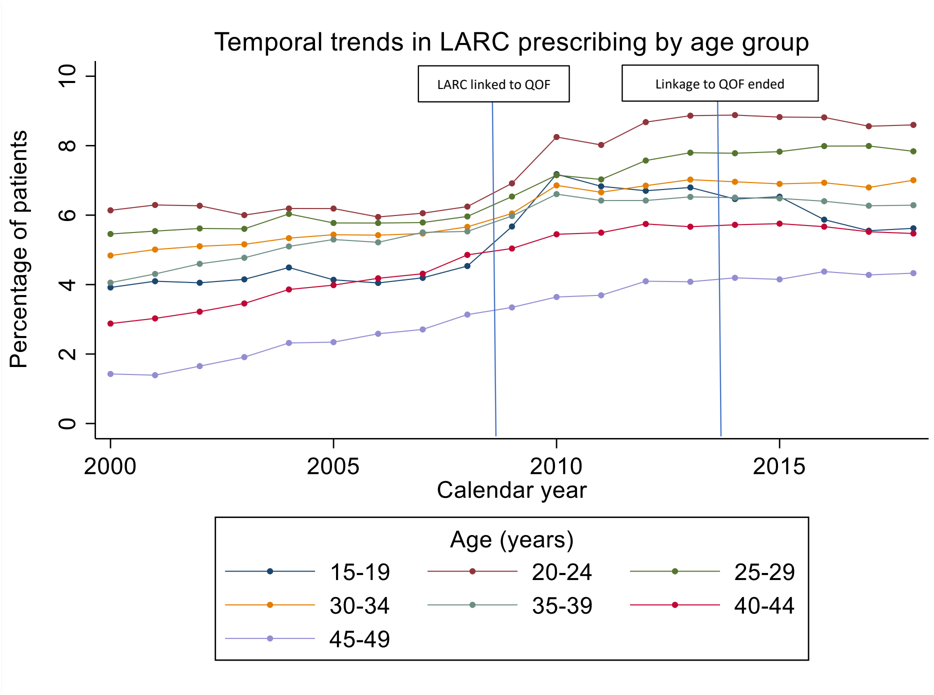

Supplement: Supplementary file 2 [file bmjsrh-2021-201260supp002.pdf]
